# A hybrid inductive-abductive analysis of health workers’ experiences and wellbeing during the COVID-19 pandemic in the United States

**DOI:** 10.1371/journal.pone.0240646

**Published:** 2020-10-26

**Authors:** Rachel Hennein, Sarah Lowe

**Affiliations:** 1 Department of Social and Behavioral Sciences, Yale School of Public Health, New Haven, Connecticut, United States of America; 2 Yale School of Medicine, New Haven, Connecticut, United States of America; University of Birmingham, UNITED KINGDOM

## Abstract

The COVID-19 pandemic puts health workers at increased risk of adverse mental health outcomes. However, no studies have assessed health workers’ experiences using qualitative methods during the COVID-19 outbreak in the United States to identify novel factors that could relate to their mental health. In May 2020, we distributed an online survey to health workers across 25 medical centers throughout the United States. The Patient Health Questionnaire-9, Generalized Anxiety Disorder-7, Primary Care-Post-Traumatic Stress Disorder, and Alcohol Use Disorders Identification Test-Concise and associated cutoff values were used to assess rates of probable major depression, generalized anxiety disorder, post-traumatic stress disorder, and alcohol use disorder, respectively. To provide insight into the factors shaping these and other mental health conditions, we included two open-ended questions asking respondents to recount their most upsetting and hopeful experiences during the COVID-19 pandemic and how it made them feel. Using a hybrid inductive-abductive approach and thematic content analysis, we created a Social Ecological Model to represent themes among health workers’ experiences within five ecological levels: individual, interpersonal, organization, community, and public policy. Of the 1,132 participants who completed the survey, 14.0% had probable major depression, 15.8% probable generalized anxiety disorder, 23.1% probable post-traumatic stress disorder, and 42.6% probable alcohol use disorder. Individual level themes included participants’ personal health and self-care behaviors. Interpersonal level themes included the health of their social circle, family functioning, and social support. Organization level themes included their hospital’s management, resources, patient care, routine, and teams. Themes in the community level included the media, scientific knowledge about COVID-19, morale, behavior, and support of health workers. Lastly, government and health system leadership and shelter-in-place policy were themes within the public policy level. Our findings provide insights into novel factors that have impacted health workers’ wellbeing during the COVID-19 pandemic. These factors should be further explored to inform interventions and public policy that mitigate mental health morbidities among health workers during this and future outbreaks.

## Introduction

In December 2019, the 2019 novel coronavirus was first identified in Wuhan, China and rapidly spread across the world [1]. On January 19, 2020, the first United States (US) case of the coronavirus disease 2019 (COVID-19) was diagnosed in Washington state [2]. Since then, there have been over 6.3 million cases and 189,000 deaths in the US as of this writing, making the US the most impacted country during the pandemic [3].

The COVID-19 pandemic, along with previous pandemics, has put health workers (HWs) at increased risk of stress, anxiety, insomnia, and depression [4, 5]. For example, a recent systematic review and meta-analysis of 13 studies found that the pooled prevalence of anxiety, depression, and insomnia among HWs in China and Singapore during the COVID-19 pandemic were 23.2% (95% CI: 17.8–29.1), 22.8% (95% CI: 15.1–31.5), and 34.3% (95% CI: 7.7–69.2), respectively [5]. Although these studies have provided insight into the mental health impact of COVID-19 on HWs, additional studies are warranted within the US, especially given the high rates of transmission within the US and the risk of additional waves of outbreak.

When attempting to target HWs at risk for poor mental health outcomes, it is important to understand the social determinants and risk factors for these mental health conditions [6]. For example, a survey study including 4,357 HWs in China found that their main concerns were family members and colleagues getting infected with COVID-19 [7]. Other cross-sectional studies in China found that perceived social support was associated with better mental health outcomes during the COVID-19 pandemic [8, 9]. However, these studies employed close-ended questions and pre-determined scales for hypothesized risk factors, and further research is needed to identify additional factors that negatively and positively impact HW wellbeing inductively.

To fill these gaps in the literature, we conducted a mixed-methods survey at 25 academic medical centers throughout the US using an online survey platform to assess HWs’ experiences in and out of the hospital and how this could impact their wellbeing. To give insight into the psychological burden of the current outbreak on HWs’ mental wellbeing within our sample, we assessed levels of major depression (MD), generalized anxiety disorder (GAD), post-traumatic stress disorder (PTSD), and alcohol use disorder (AUD). Through analysis of two open-ended questions asking about HWs’ most upsetting and most hopeful experiences during the COVID-19 pandemic, we created a Social Ecological Model to understand the context of HWs’ wellbeing. This information could inform primary and secondary prevention interventions and public policy to mitigate the mental health consequences of the COVID-19 pandemic on HWs during the current and future disease outbreaks.

## Methods

### Setting

This study is part of a larger cross-sectional study of HWs’ psychosocial functioning across the US. During May 2020, we distributed an online, cross-sectional survey to HWs at 25 medical centers across the US. During this period, the total confirmed cases of COVID-19 peaked in the US, exceeding 1.74 million cases and 100,000 deaths [10]. Hospitals were chosen based on their geographic location to include HWs in the Northeast, Midwest, South, and West regions of the US. The Yale Institutional Review Board approved the study, and participants provided informed consent. The 32-item checklist from the Consolidated Criteria for Reporting Qualitative Research was employed to direct study reporting [11].

### Eligibility and recruitment

We employed convenience sampling whereby hospital department chairs and division chiefs were contacted at each hospital to forward our recruitment email and online survey to their staff. The recruitment email stated that our purpose was to understand HWs’ experiences within and outside the hospital during the COVID-19 pandemic. Individuals were eligible if they were at least 18 years of age and were HWs. HWs were defined as those who work in a hospital or clinic, including but not limited to attending physicians, medical trainees (i.e. residents and fellows), physician assistants, nurses, medical assistants, nurse assistants, research coordinators, social workers, administrators, lab technicians, health technicians, psychologists, and respiratory, occupational, and physical therapists.

### Data collection

Demographic data collected included: age, gender, race/ethnicity, marital status, occupation, and geographic location. One question was used to ascertain HWs’ front line status, i.e. if they worked directly in COVID-19 rooms.

The survey included previously validated scales for mental health outcomes. These patient-reported outcome measures were included to give context of the psychological burden of the pandemic in the sample. The Patient Health Questionnaire-9 (PHQ-9) [12, 13], Generalized Anxiety Disorder-7 (GAD-7) [14], Primary Care-PTSD (PC-PTSD) [15, 16], and Alcohol Use Disorders Identification Test-Concise (AUDIT-C) [17] were used to assess MD, GAD, PTSD, and AUD, respectively. The cutoff values for probable clinical depression, GAD, and PTSD are PHQ-9> = 10, GAD-7> = 10, and PC-PTSD> = 3, respectively. For AUDIT-C, a score of 4 or more is considered probable AUD for men and a score of 3 or more is considered probable AUD for women. Patient-reported outcome measures and risk factors will be further explored in a separate study.

To assess HWs’ unique experiences during the COVID-19 pandemic, we included two open-ended questions asking respondents to recount their most upsetting and most hopeful experiences during the COVID-19 pandemic and how it made them feel. We chose these two questions based on the literature that has demonstrated substantial variability in psychological responses to trauma, including severe and persistent trauma-related symptoms and experiences of psychological growth [18, 19]. A meta-analysis of 63 studies including 26,951 patients found that trauma survivors sometimes experience both post-trauma symptoms and psychological growth simultaneously [20]. We therefore asked two-open ended questions–one focusing on negative experiences and another focusing on positive experiences–to understand the range of factors that account for such variability in psychological outcomes within and between participants. Mixed methods studies have used similar approaches, including assessing positive and negative experiences among survivors of Hurricane Katrina [21, 22].

Our survey was piloted in two iterations with one attending physician and one resident physician to receive their feedback regarding appropriateness of questions, interpretation of the open-ended questions, and survey length. Their responses were excluded from our analysis.

We defined data saturation as the point at which new themes were no longer emerging in subsequent responses [23]. Data saturation was reached after 50 responses. However, to better understand the frequencies of the positive and negative experiences among the sample, we continued to code all available responses.

### Analysis

Responses to both open-ended questions were coded in two rounds by one researcher (RH, MD-PhD candidate, six years of qualitative analysis experience, female), and the code tree was reviewed and validated by another researcher (SL, MA, PhD, Assistant Professor and Clinical Psychologist, Trauma and Mental Health content expert, over decade of qualitative analysis experience, female). Both researchers reviewed and discussed sample quotes during the coding process to ensure that code definitions were consistent and appropriately applied to participant responses. Using an inductive approach, codes were first developed that represented themes that were present across responses [24].

Once the code tree was finalized after two rounds of inductive analysis, codes were grouped using an abductive approach [25] based on the Social Ecological Model and analyzed using thematic content analysis [26]. The Social Ecological Model holds that an individual’s mental wellbeing and behavior is influenced by interactions of individual, interpersonal, organization, community, and public policy levels [27–29]. The individual level includes factors related to their attitudes, beliefs, knowledge, and behavior. Factors within the interpersonal level include families, friends, and social networks. The organization level includes factors related to their workplace and businesses, which largely focused on the hospital or health system in which the respondents work for our study. Community level factors relate to the culture and communications within the population. Lastly, public policy level factors include interactions with the national, state, or local government and policies.

To give an example of our analysis process, inductive analysis was employed that identified various codes, including ‘childcare changes,’ ‘homeschooling,’ ‘household finances and job security,’ and ‘familial bonds.’ Using thematic content analysis, we grouped these codes within a single theme called ‘Family Functioning.’ Using an abductive approach and the Social Ecological Model, we mapped the ‘Family Functioning’ theme to the interpersonal ecological level.

Qualitative responses were coded using Microsoft Excel to facilitate integration of responses and codes with the quantitative dataset. Quantitative analysis was performed using SPSS statistical software (IBM Corp). We computed mean scores for each mental health indicator in the overall sample. We also determined the prevalence of probable MD, GAD, PTSD, and AUD in the sample using the pre-defined cutoff values. Assessing risk factors for mental health conditions using multivariable logistic regression was beyond the scope of the current study; however, we aim to conduct those analyses in a separate paper.

## Results

### Sample

A total of 1,132 participants completed the survey. Descriptive information on participants is provided in Table 1. Most respondents were female (71.4%), were married (64.4%), and identified as White (74.6%). Of the 1,132 respondents, 31.3% were physicians, 17.6% were medical trainees, 18.9% were nurses, 5.9% were physician, nursing, or medical assistants, 8.1% were health technologists or technicians, 8.2% had another clinical job, and 9.7% had a non-clinical job. About half of the respondents participated in direct care with patients with COVID-19 (51.9%). Most respondents were from the Northeast region of the US (53.7%), followed by Midwest (23.7%), West (12.7%), and South (9.5%). The average scores for depression, GAD, PTSD, and alcohol use disorder were 4.8 (95% CI: 4.5–5.1), 4.9 (95% CI: 4.6–5.2), 1.4 (95% CI: 1.4–1.5), and 2.5 (95% CI: 2.4–2.6), respectively. Based on the cutoff values for the mental health scales, 14.0% of the sample had scores indicative of probable MD, 15.8% probable GAD, 23.1% probable PTSD, and 42.6% probable AUD.

**Table 1:** Participant characteristics.

Characteristics	
Overall N	1132
**Gender, N (%)**	
Women	808 (71.4)
Men	320 (28.3)
Non-binary	3 (0.3)
Other	1 (0.1)
**Race/ethnicity, N (%)**	
Asian	147 (13)
American Indian or Alaska Native	4 (0.4)
Black	53 (4.7)
Hispanic	62 (5.5)
White	845 (74.6)
Native Hawaiian or Pacific Islander	2 (0.2)
Other	21 (1.9)
**Marital status, N (%)**	
Married	729 (64.4)
Unmarried	403 (35.6)
**Occupation, N (%)**	
Physician	354 (31.3)
Medical trainee	199 (17.6)
Nurse	214 (18.9)
Physician, nursing, or medical assistant	67 (5.9)
Health technologist or technician	92 (8.1)
Other clinical job	93 (8.2)
Non-clinical job	110 (9.7)
Other, not specified	3 (0.3)
**Front line, N (%)**	
Second line	345 (30.5)
Front line	588 (51.9)
**Region, N (%)**	
Northeast	608 (53.7)
South	107 (9.5)
Midwest	268 (23.7)
West	144 (12.7)
**Mental health scores, Mean (SD)**	
PHQ-9	4.8 (4.6)
GAD-7	4.9 (4.8)
PC-PTSD	1.4 (1.3)
AUDIT-C	2.5 (1.8)
**Probable mental health disorder, N (%)**	
Major depression	159 (14.0)
GAD	179 (15.8)
PTSD	262 (23.1)
Alcohol use disorder	482 (42.6)

### Social ecological model

**Fig 1:**
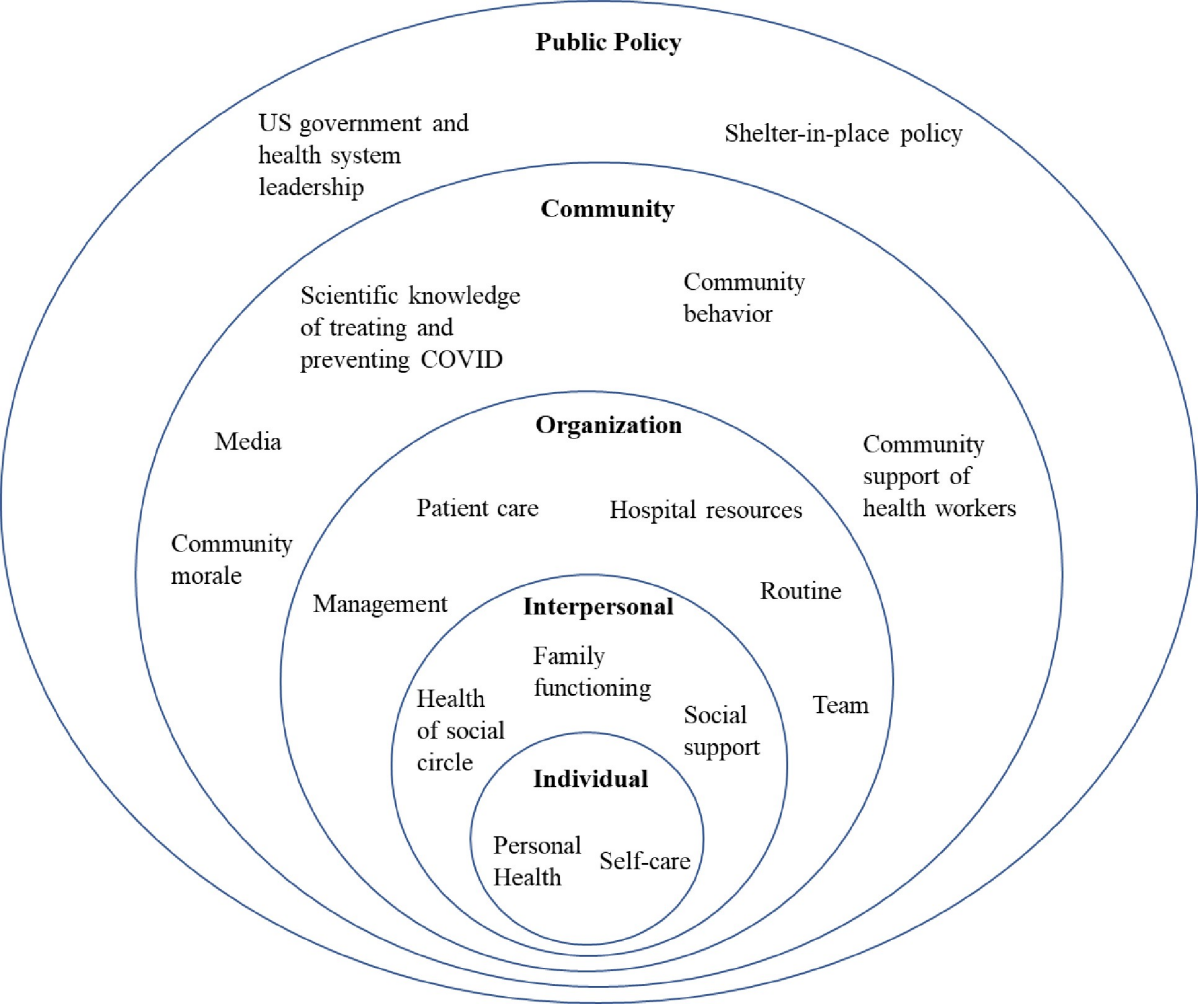
Social ecological model for health workers’ wellbeing during the COVID-19 pandemic.

Thematic content analysis revealed individual-, interpersonal-, organization-, community, and public policy-level themes that impact HWs’ negative and positive experiences in and out of the hospital. Fig 1 organizes these themes within a Social Ecological Model. Table 2 depicts these themes, subtheme factors, and representative quotes organized by social ecological level.

**Table 2:** Social ecological model themes, factors, and associated quotes for the most upsetting and hopeful experiences reported by health workers during the COVID-19 pandemic.

Theme	Factor	Upsetting or hopeful	Quote
**Individual level**			
**Personal health**	COVID infection	Upsetting	Patient coding [in cardiopulmonary arrest] during intubation, she was only 52, obese, difficulty to oxygenate and ventilate, lost my visor during CPR, got her back and intubated, but was definitely exposed. Got sick with COVID 5 days later, infected my husband despite quarantining (female, 51 years old, physician, front line worker, South)
		Hopeful	Was tested for COVID 19 and came back negative. Felt much more at ease since then. (female, 66 years old, administrative assistant, second line worker, Northeast)
	Accessing non-COVID medical care	Upsetting	we discovered I was pregnant Feb 29th before the pandemic hit. Exploring our options and navigating a pregnancy through a pandemic is hard, especially when you have to go to your appointments alone and not receive the care you need through a virtual appointment. (female, 26 years old, medical assistant, second line worker, Midwest)
		Hopeful	signing up for therapy for the first time in my life; it has been a great addition where I get an hour to talk about whatever I want and how it affects me (female, 28 years old, medical trainee, front line worker, Midwest)
**Self-care behaviors**	Time to process stress	Upsetting	There was a day in which I supervised residents who had to pronounce death five times in 3 hours. It was a lot, and there were a lot of tears from them for understandable reasons. But what was hard was that I then had to go join planning Zoom calls in which no one has a transparent plan. And I had to go home to take care of my three kids. there is no room for some of us to process this, because no one will help us find time in our lives to process it. (female, 39 years old, physician, front line worker, Northeast)
	Self care activities	Hopeful	For the first time in my residency training I've had the time to reflect on the meaning of my job of being a resident and what I value about it. I've had more time for family as well. And plenty of time to eat well and exercise which I don't usually have during covid. These self care techniques allow me to appreciate the role of resident and trainee in a way I found hard when I felt our work hours were too demanding. (female, 29 years old, medical trainee, second line worker, Northeast)
**Interpersonal level**			
**Health of social circle**	Fear of infecting family with COVID	Upsetting	With regard to most upsetting part of working with COVID-19 patients, I cannot get the image of my young son (4 years old) running towards me to hug me after coming home from my shift when the pandemic first hit my hospital. I yelled at him to stay away and my husband had to run and grab him. The look of sheer confusion and disappointment on his face has stayed with me all of these weeks. I cannot get that image out of my head. He now understands I need to shower before hugging him. (female, 40 years old, nurse, front line worker, Northeast)
	COVID infection or death	Upsetting	A good friend died from COVID-19 and I became infected with the virus. I am still grieving over the loss of my friend and it took about a month to recover completely. (female, 59 years old, health technologist, second line worker, Midwest)
	Compliance with COVID safety precautions	Upsetting	Having friends and family not take the pandemic seriously is upsetting. I am telling family members daily that their loved one has died alone in a hospital room. Seeing them go about their daily lives without masks or consideration for those who they might kill, or that they might get sick themselves, is infuriating. This, and the general state of the country at large make it difficult to keep my morale up. It feels like drowning. (Female, 28 years old, medical trainee, front line worker, South)
		Hopeful	My son started wearing his masks and I found out that 2 cousins were recovering from Covid19 (female, 59 years old, health technologist, second line worker, Midwest)
	Medical care unrelated to COVID	Upsetting	My parents 89 yrs and 96 years are isolated both in separate locations as my mom got sick and needed hospitalization (non covid related). I have been banned from seeing them due to Covid positive patients where I work. (female, 59 years old, nurse, second line worker, Midwest)
		Hopeful	My wife’s pregnancy has been going well and we are making plans for his arrival. (male, 33 years old, medical trainee, front line worker, West)
**Social support**	Physical isolation	Upsetting	Sending my baby away and not being able to see her daily, and getting sick coming down with covid19 symptoms that made me sick for two weeks without seeing my child. I felt isolated and useless. (female, 36 years old, nurse, second line worker, Midwest)
	Receiving social support during isolation	Upsetting	I am very close to my family and they are a large part of my support system. It was very hard for me to deal with the increased stress of work and have less outlets and positive interactions with friends and family. (female, 25 years old, nurse, first line worker, Northeast)
		Hopeful	friends, neighbors, and others dropped off or sent masks and other PPE to my house without my ever asking. it made me feel supported as a healthcare worker and more confident in our ability to meet the need for more PPE. (female, 37 years old, physician, first line worker, Northeast)
	Thankfulness	Hopeful	So many of my friends and loved ones have made an effort to thank me for the work that I am doing. I am very lucky to have friends that live all over the world, and to receive little care packages form people I miss regardless has been very encouraging and uplifting. I have felt immense love. (female, 25 years old, health technician, first line worker, Northeast)
**Family functioning**	Childcare	Upsetting	Not being able to get around my parents. Having to balance still working with kids at home. Finding someone to oversee watching the children since daycare's closed and still have not opened. Making sure homework was still getting done. Overwhelmed. Overworked between career job and motherhood. (female, 42 years old, nurse, second line worker, South)
	Family stress	Upsetting	My 11 year old son broke down crying because he was concerned about my wife and myself because we are both doctors and might get exposed /sick, he was tired of hearing about COVID "everywhere", and he felt like "everyday was the same" from being stuck at home. It made me sad that he was dealing with this and that despite my efforts to help him, my other responsibilities meant that I didn't do enough. (male, 50 years old, physician, front line worker, South)
	Bonding	Upsetting	My husband and I are both internal medicine residents, working in direct COVID care. In March, as the pandemic in the US was rapidly worsening, we made the difficulty decision to live separately from our 2 year old son and my mother, who helps us take care of him. We were unable to provide for his childcare on our own since we were both working, and we felt very worried about transmitting the coronavirus to my mother, who is in her late 60s. It has been devastating to live apart from him: As residents and parents, we’ve spent the last two years trying to optimize every free minute with our son. In normal times, I worry constantly about being an adequate parent. Now, I fear that his security in knowing he is loved and the bonds we’ve worked so hard to foster are being broken down by this time apart. (female, 33 years old, medical trainee, front line worker, Northeast)
		Hopeful	My family has been able to slow down our lifestyle. We were constantly on the go. Now we spend more time outdoors at home. My yard has never looked better. I like to spend time outdoors where it is peaceful. Our pets are so happy to have us around all this time and we are enjoying their constant companionship. I am cooking more meals at home and we are eating healthier. It feels great to lead a less chaotic lifestyle. (female, 47 years old, health technologist, front line worker, Northeast)
	Finances	Upsetting	Financial hardship as I became the only employed adult in our home of 5 adults. (female, 24 years old, health technician, second line worker, Midwest)
		Hopeful	Deciphering the importance of basic needs and materialistic wealth. Paying off my debt since I'm not going anywhere and spending money. (female, 32 years old, nurse, second line worker, Northeast)
**Organization level**			
**Hospital resources**	PPE	Upsetting	Not being given proper PPE. It made me feel as if they didn't care if I contracted Covid (female, 59 years old, nurse, second line worker, South)
	Human resources	Upsetting	The only RN on our unit retired. We have been told that we cannot fill the position right away due to mass RN hiring related to COVID. We are very short staffed and other work is being put on hold or de-prioritized due to COVID. (female, 44 years old, nurse, second line worker, West)
	Testing	Upsetting	Not being able to perform tests on patients I was concerned about having COVID-19 because of the lack of testing. It makes me feel upset with the federal government and the total lack of leadership from the President and his associates. (male, 59 years old, physician, front line worker, Midwest)
		Hopeful	patients being screened for covid-19 prior to admission to my floor (male, 54 years old, nurse, front line worker, Northeast)
**Hospital routine**	Work hours	Upsetting	I have changed shift times, location, and role several times in the past 2 months. . .and forced to use vacation time given threat of being redeployed to a COVID unit. Makes me feel like a number and not a person. (female, 38 years old, physician assistant, front line worker, Northeast)
		Hopeful	The most positive thing has been less workdays/ less weekly duty hours in the hospital. This has made me feel happy and less burnt out! (male, 30 years old, medical trainee, front line worker, South)
	Duties	Upsetting	Being redeployed to work outside of my specialty. Working on covid floors and medically managing sick patients worried me. Am I going to be able to recognize if a patient takes a turn for the worse, am I going to know what to do? Will I make the right decision with little to no training? At times i felt overwhelmed and unsure of myself. Did I remove PPE properly, did I touch my face? Will I bring home anything and get my children or husband sick? (female, 41 years old, physician assistant, front line worker, Northeast)
		Hopeful	My interest in my job has been completely rejuvenated, I have never been more excited to be a doctor. It is a privilege to be in the role. This is a once in a lifetime challenge and I want to step up to that challenge. (male, 29 years old, medical trainee, front line worker, Midwest)
	Wearing PPE	Upsetting	I led a trauma resuscitation of a 5 year old boy and while we were all properly PPE'd I feel that all the thought and prep that goes into making sure we're all safe and minimizing personnel exposure, we may be sacrificing the same quality of preparation and care that would normally be given to a critically ill or injured child. We lost pulses on the child almost immediately upon arrival to the our trauma bay and we attempted to resuscitate him for almost an hour until his father arrived and then we stopped resuscitative efforts. The communication between staff in the bay and our newly formed command center is difficult due to masks and face shields and some in PAPR. (female, 41 years old, physician, front line worker, Northeast)
	Teleheatlh	Upsetting	I was forced into doing video visits with patients that I was ill prepared to do. I was never able to get training because of the backlog and felt forced into doing something I was not competent enough to do. I fear having to take time in the future to correct mistakes or do additional work to compensate for this that will need to be done after hours. (female, 50 years old, social worker, second line worker, Midwest)
		Hopeful	My mental health has significantly improved. I am able to work from home and feel more content, at peace, relaxed, and more focused on my work as a result. I recognize that I am one of the few and lucky. Also, I have no fear of becoming infected and/or getting sick. (female, 29 years old, social worker, second line worker, Midwest)
**Patient care**	COVID patient outcomes	Upsetting	While working at my other job as an EMT, I had a covid patient around my age. He was extremely sick. His children, about the same age as mine, carried their father outside to the ambulance. I could see the worry and fear on their faces. After assessing the patient, he had an o2 sat of 58%, it was clear that his chances of survival were grim. The children became aware that they too had been exposed to the virus, which was also terrifying to them. We took care of the patient's immediate medical needs and prior to leaving for the hospital, my partner looked at the family and said, "you have two minutes to say whatever you need to your dad. He will more than likely be intubated immediately upon arriving at the hospital." At this point, everyone was aware of what that meant, say your good byes now, because it may be your last chance. The kids and wife were crying. Things were moving quickly. No family members were allowed to come with the patient to the hospital. This would more than likely be the last time they would see him alive. It was heart breaking. In 30 years as an EMT, this felt different than any other similar call I done. Normally as we leave, the family is hopeful that the patient can be saved. They can be with the patient and say their goodbyes at the hospital. They can grieve at the patient funeral services. NOT in the back of my ambulance. As we pulled out of the patients driveway, we left his entire family sitting in front of their house grieving, scared, wanting to believe there was a possibility we could save their father, knowing there was a good chance that that was the last time they would see him alive. Scared for themselves on so many level. The last image I have of this mans family is of them all in the driveway crying. The patient was intubated immediately, and did die a few days later. He had been in good health prior to covid. He was almost the same age as myself. His children were the same age as mine. (female, 50 years old, health technician, front line worker, Northeast)
		Hopeful	Our unit was able to provide ICU care along with ECMO support for a young, pregnant patient who tested positive for COVID. She made it off ECMO and is now at home recovering (baby is okay too!). (female, 29 years old, nurse, front line worker, Midwest)
	Isolated patients	Upsetting	Patient family members aren’t allowed to visit COVID positive patients even if they are dying or have deceased. Patients die alone and it is traumatic to watch. (male, 31 years old, nurse, front line worker, Midwest)
		Hopeful	I really enjoyed bringing families to morning bedside rounds via phone or FaceTime. It made me and the team feel very connected to the patients and their families. It allowed me to evaluate how the House Staff informs patients with their family members present of their plan and the pathophysiology of their disease in lay terms. It helped families stay connected while they couldn't visit personally. I'd like to keep this practice part of daily rounds going forward. (female, 50 years old, physician, second line worker, Northeast)
	Non-COVID medical care	Upsetting	Several of my primary care patients have died from non-covid related causes. They were patients with significant co-morbidities who were hesitant to come to the hospital early for evaluation. (female, 53 years old, physician, second line worker, Midwest)
	Patient and family appreciation	Hopeful	Patient called me his “lifesaver” when I discovered his pulmonary embolism. A family member told me I personally helped make her husband’s unfortunate death peaceful for them. Family members of patients thank me and tell me they are praying for my safety. All of these stories make it worth it, and bring me some hope and motivation to keep going. (female, 29 years old, medical trainee, front line worker, Northeast)
**Hospital team**	Colleague diagnosed with COVID	Upsetting	Having a colleague and friend as a patient and admitting him to the ICU. We are similar in age, hobbies, healthy lifestyles and life responsibilities. It made me feel sad, angry and scared on how this could happen to all of us. (male, 41 years old, research staff, front line worker, South)
	Teamwork	Upsetting	Lack of participation in caring for these patients. Being repeatedly exposed to high risk situations because others don’t want to. Makes me very angry (male, 39 years old, nurse, front line worker, South)
		Hopeful	Witnessing the team work and camaraderie. It is wonderful to see people pulling together (mostly). It reminds me of my time in the military when there were shared experiences, a common enemy and coming together to overcome adversity. (male, 68 years old, physician assistant, second line worker, Northeast)
	Emotional support	Hopeful	increased friendships with colleagues-more personal connection, more vulnerability with each other makes me feel closer in some ways. (non-binary, 37 years old, psychologist, second line worker, West)
	Inspiration	Hopeful	hearing the stories of what some of my colleagues have done to help families in inspiring ways and stories of other people getting together to help each other (male, 31 years old, medical trainee, front line worker, Northeast)
	Supervisor relationship	Upsetting	Feeling as though my supervisor has not been open or sensitive to feedback about current workload and equal sharing of work among our team. (female, 35 years old, physician assistant, front line worker, Northeast)
		Hopeful	Positive feedback from supervisors (male, 33 years old, medical trainee, front line worker, Northeast)
**Hospital management**	PPE policies	Upsetting	I was infected with COVID 19 after being floated from my ortho floor to a COVID floor. I was told to rewear and disinfect disposable PPE several times. I never felt safe at my own job. I verbalized numerous times my concern for my safety to the team and middle management. I felt taken advantage of, disposable, like I didn't matter. I stayed in my bedroom for 11 days as my family was terrified of my illness. I did everything I could to prevent being sick. I changed before leaving work, cleaned everything down, brought disposable products (water bottles, etc.) to work. I still got infected. I go to work and they act as if nothing happened to me. It makes me feel unvalued by my organization. I thank God for my health now. I just feel defeated. (male, 25 years old, nurse, front line worker, Northeast)
		Hopeful	When the system responded and dramatically changed the hospital policies to protect its providers, it gave hope that we would come out of this pandemic. (female, 33 years old, medical trainee, second line worker, Northeast)
	Leadership	Upsetting	Hospital Leadership—both administration, medicine, and nursing, has devalued nursing by their communications and actions. Despite vocalizing they are 'thankful' for all of us they demanded everyone be relocated to unfamiliar units for COVID, stating that at a moment's notice we would also have to staff a field hospital. Now they are cutting staffing, cutting budgets despite the fact our emergency service is seeing a surge of high acuity patients with no where to place them (long lengths of stay). And not to mention the constant change in policies and the absolute minimum protection of PPE. All of this is very disturbing and makes me want to leave the profession (female, 40 years old, nurse, front line worker, Midwest)
		Hopeful	My hospital has actually been great and it is been helpful. Daily a message was released about COVID+ numbers and discharges, they have given time for working parents to parent their children (who are now home) and they just released a training to get people back to work that included information on wearing masks, one-way hallways, etc. It has been positive and science driven (female, 45 years old, social worker, second line worker, Midwest)
	Job security	Upsetting	Working 24/7 on urgent initiatives because there is a fear of layoffs in the near future and we need to get things done while we still have resources, being treated as though I'm not doing enough, and simultaneously being told to be sure to take care of myself and my mental health. It makes me feel frantic and disposable. (female, 32 years old, administrator, second line worker, West)
	Modified schedules	Hopeful	The schedule change that was put into place to protect part of the work force served to indicate that my superiors were trying to protect us (and maintain a backup work force). Honestly it was also nice to have a couple days not at the hospital. Which wouldn't have been the case during this time of year under normal circumstances. I got to go on walks outside in the sun with my dog. I don't normally get to do that. (male, 29 years old, medical trainee, front line worker, West)
	Hazard pay	Hopeful	Hazard pay and acknowledgment of the costs the pandemic has placed on us (male, 32 years old, medical trainee, front line worker, Northeast)
**Community level**			
**Community behavior**	Protesting shelter-in-place	Upsetting	The most upsetting thing to see is people protesting about their perceived loss of freedom related to stay at home orders and being asked to wear masks. (female, 42 years old, physician, second line worker, South)
	Compliance with COVID safety precautions	Upsetting	seeing careless people ignore safety directives—made me feel like our sacrifices in the frontlines (personally and to my family) were unappreciated and dismissive of patients suffering/losses (female, 41 years old, physician, second line worker, Northeast)
		Hopeful	The vast majority of people following social distancing, shelter-in-place and mask wearing recommendations. Makes me feel proud. (male, 54 years old, physician, front line worker, South)
**Media**	Misinformation	Upsetting	The juxtaposition of working to fight a pandemic all day and then spend my free time fighting an infodemic among family members, social contacts (online), and the media. I am exhausted by the amount misinformation being believed over my professional opinions and experience. (female, 37 years old, medical trainee, front line worker, West)
	Positive news	Hopeful	Positive news stories about people pulling together to help and uplift others. Definitely raises your mood levels and gives you hope for the future. (female, 64 years old, nurse assistant, second line worker, Midwest)
**Community Morale**	Discrimination against people of Asian descent	Upsetting	Increased/blatant racism towards healthcare workers of Asian descent (male, 29 years old, medical trainee, front line worker, Northeast)
	Fear	Upsetting	Neighbors fearing neighbors. FEAR. People are afraid of everything. Politics and this pandemic are tearing our country further apart. (female, 37 years old, health technologist, front line, Northeast)
	Community altruism	Hopeful	Seeing people in my community donating money to help our neighbors who need food and supplies. (female, 53 years old, social worker, second line worker, Midwest)
		Hopeful	The overall community response of neighbors reaching out to each other and supporting our small business has been really touching (female, 27 years old, physician assistant, second line worker, West)
**Community support of health workers**	Conspiracy theories	Upsetting	The most upsetting thing has been reading in the media that there are folks out there who think this is a hoax and that we are making it up in order to receive monitary benefits from the government. We are putting our lives at risk every day we come to work and people are thinking there is a secondary gain. It makes me feel so unappreciated. It also makes me fear that people like that will be more likely to take risks and have higher potential to pass this virus onto others. (female, 46 years old, physician assistant, front line worker, Northeast)
	Stigmatization of health workers	Upsetting	i had gone to a target after work in my scrubs and everyone i walked by looked at me like i had the disease. i have never been looked at and felt like people were afraid/disgusted with me (female, 22 years old, health technologist, front line worker, Northeast)
	Recognition of health workers	Hopeful	Neighborhood kids chalked 'Thank you Doctor XXXX and Doctor XXXX' on our driveway. Was during the busiest/hardest time during the outbreak, and it meant a lot. Still get somewhat emotional thinking about it. (male, 42 years old, physician, second line worker, Midwest)
	Donations to health workers	Hopeful	Seeing people donate PPE to the hospital. It made me feel like we are all a team. (female, 37 years old, physician, front line worker, Midwest)
		Hopeful	all of the donations of food/supplies/cards to me personally or the ED for the work we do every day. it makes us feel like we are not alone and being seen. (female, 33 years old, nurse, front line worker, Northeast)
		Hopeful	Receiving food from local restaurants at the hospital—makes me feel appreciated (female, 30 years old, medical trainee, front line worker, South)
**Scientific knowledge of treating and preventing COVID**	Uncertainty	Upsetting	The fear of the unknown has been the most upsetting thing that has happened to me. Unsure of how the disease was transmitted at the start, unsure if we would have enough PPE, unsure if we would be laid off, unsure if we would die. (female, 26 years old, nurse, front line worker, Northeast)
	Hope for a cure	Hopeful	The most positive thing is knowing that there will soon be a cure for this virus. (female, 37 years old, health technician, second line worker, South)
	Hope for a vaccine	Hopeful	knowing that scientists are working on a vaccine. (female, 29 years old, medical trainee, front line worker, Midwest)
**Public Policy level**			
**General US government and health system leadership**	Lack of national response	Upsetting	It isn't one thing specific. I am upset with the response and hearing everyone's opinion. The lack of accountability from the federal government, the lying from all leaders, the CDC changing language just to protect themselves. It feels like we're all being lied to. And due to that, everyone is trying to figure it out for themselves which is causing an explosion on social media, heightened emotions, anger, fear. It's just frustrating and greatly impacting morale at work. The little men look to the leaders for answers and a direction, and they're a fucking [sic] mess and it leaves us just to figure it out. Pardon my language. But you get it. (female, 27 years old, nurse, front line worker, Northeast)
	Lack of scientific-driven policy	Upsetting	The lack on clear nation leadership and the ongoing use of divisive politics. The absence of scientifically based policy is a national disgrace. (male, 67 years old, physician, second line worker, Northeast)
	Misinformation	Upsetting	THE PRESIDENT OF THE UNITED STATES, he is destroying our country, suggesting people should inject bleach or Lysol. He has no regard for life, except his own. I am fearful for our country. (female, 66 years old, nurse, second line worker, South)
		Upsetting	I have had to start taking overnight call again assignment to intubate all COVID patients and line them up (3 times in last month). Sleep and worry very poor these nights. NOT to be political but the lies that come out of the White House make my job more difficult and I am concerned about a resurgence when we already have had to treat 2 ICU units housing 30 COVID patients. The public just doesn't seem to get it. Last night driving home there was a picketer outside the hospital dressed in a Scream outfit with a sign "Resist Medical Tyranny" ugh (female, 59 years old, physician, front line worker, Midwest)
	Opportunity to improve policy	Hopeful	I feel that the health care delivery in the United States is finally getting attention. Perhaps this will be an incentive to improve health care coverage and delivery to those who need it. (male, 67 years old, physician, second line worker, South)

Within the individual level, the respondent’s health status and ability to practice self-care behaviors were themes present in both the most upsetting and most hopeful answers. In the interpersonal level, respondents reported that social support, the health of their social circle, and family functioning were themes that related to both their positive and negative experiences during the pandemic. In the organization level, themes identified that relate to the negative and positive experiences of the HWs include hospital resources, routine, management, team, and patient care experiences. In the community level, availability of scientific knowledge about COVID-19, compliance with social distancing, community morale, support of HWs, and the media were themes related to the positive and negative experiences of the respondents. Lastly, in the public policy level, the US government and health system leadership and shelter-in-place policy were themes that contributed to the positive and negative experiences of the HWs.

### Individual level themes

Among the sample, 40 (3.5%) and 124 (11.0%) respondents reported that their most upsetting and hopeful experiences, respectively, occurred in the individual level. Individual level themes pertained to the HWs’ health status and ability to practice self-care behaviors. Within the personal health theme, factors included COVID-19 infection and accessing non-COVID-19 medical care. Regarding COVID-19 infection, many respondents expressed that getting infected with COVID-19 was their worst experience during the pandemic. Other HWs reported that when they tested negative for COVID-19, they were hopeful and were put “at ease.” The respondent’s health status and ability to pursue medical care for medical conditions not related to COVID-19 also contributed to their most upsetting and hopeful experiences. For example, difficulties accessing obstetric, surgical, and physical rehabilitation care were among the most upsetting experiences for some HWs. On the other hand, many respondents suggested that beginning psychotherapy was their most hopeful experience during the pandemic, as it improved their mental health.

Another theme in the individual level was HWs’ ability to pursue self-care behaviors, which included two factors: time to process stress and self-care activities. Some respondents suggested that their work hours had increased without additional days off, which prevented them from engaging in self-care behaviors and process the added stress of the pandemic. For example, one physician recounted when she “supervised residents who had to pronounce death five times in 3 hours,” without the ability to fully cope with the experience “because no one will help us find time in our lives to process it.” Regarding hopeful experiences, others recounted that having more time off from clinical duties enabled them to have more time for spiritual practices, hobbies, exercise, healthy cooking, and self-reflection.

### Interpersonal level themes

Next, 437 (38.6%) and 352 (31.1%) respondents suggested that their most upsetting and hopeful experiences, respectively, occurred within the interpersonal level. For example, respondents expressed that the health of their immediate social circle, including family members and close friends, contributed to their most upsetting and hopeful experiences. Within the health of social circle theme, factors included: fear of infecting family with COVID-19, COVID-19 infection or death, compliance with COVID-19 safety precautions, and medical care unrelated to COVID-19. Many respondents suggested that they feared that their household members would get COVID-19 from them given their exposure at the hospital. Respondents also wrote that their most upsetting experiences were related to their family members thinking that they had COVID-19, testing positive for COVID-19, or dying from COVID-19. Many respondents were upset that their immediate social circle was not taking COVID-19 safety precautions seriously, i.e. shelter-in-place and wearing masks, or believed that COVID-19 was a “hoax.” One medical trainee expressed that it is “infuriating” to her when her family “goes about their daily lives without masks or consideration for those who they might kill, or that they might get sick themselves.” However, some respondents said that they felt hopeful when their family members were compliant with COVID-19 safety precautions. Other HWs suggested that the health of their social group unrelated to COVID-19 caused them significant distress. Many respondents were upset that they could not visit their family members who had a serious medical condition out of fear of infecting them. Others recounted hope for their social circle’s health, such as a healthy pregnancy.

The second theme within the interpersonal level related to social support, which included the factors of physical isolation, receiving social support during isolation, and thankfulness. Many HWs reported feeling upset about physical isolation during the pandemic and made them feel “useless” when wanting to help their family. Furthermore, many recounted that their ability to receive social support was altered during the pandemic. For example, many expressed that decreased interactions with family and friends due to social distance restrictions made it difficult for them to receive support during this high stress time. However, others reported that they received more virtual and social-distanced support, which took the form of letters, care packages, and video calls from their friends, family, and neighbors. Respondents also felt hope when their social circle thanked them for their work as a HW, which made one health technician feel “immense love.”

Third, family functioning was a theme within the interpersonal level and included the factors: childcare, family stress, bonding, and finances. Many HWs recounted hardships caused by the closure of childcare and schools during shelter-in-place. One nurse reported that she felt “overworked between career job and motherhood.” HWs who are parents also suggested that there was added stress within their families as their children were processing the pandemic. Respondents who had to isolate themselves from family members expressed that this negatively impacted their family bonding. One medical trainee recounted that having to isolate from her 2-year-old son made her fear that “bonds we’ve worked so hard to foster are being broken down by this time apart.” However, many respondents who were able to shelter-in-place with their family suggested that increased time together strengthened their bonds. Many suggested that they were grateful that their children’s activities were canceled so that their family could slow down and spend more quality time together. Some HWs suggested that their families were facing financial hardships due to job loss; however, others suggested that their families were able to save money during this time and focus on non-materialistic values.

### Organization level themes

Within the organization level, 457 (40.4%) and 445 (39.3%) participants reported upsetting and hopeful experiences, respectively. Five organization level themes were identified in the data, including hospital resources, routine, patient care experiences, team, and management. First, many respondents reported that the most upsetting aspect of the pandemic has been the lack of hospital resources, which includes the subtheme factors of personal protective equipment (PPE), testing, and human resources. Respondents suggested that the lack of essential resources made them feel unsafe, dehumanized, and “as if they didn’t care if I contracted COVID.” On the other hand, some respondents suggested that increased testing contributed to their hope.

Many respondents reported that there was a change in their hospital routine due to the pandemic, including changes in work hours, schedule, duties, wearing PPE, and work settings. For example, increased workload and hours were reported as being the most upsetting aspect of the pandemic, while decreased workload and hours were reported as being the most hopeful. Many respondents also reported that they felt distressed when their duties shifted to provide care to units outside of their specialty with minimal training and resources, which made one physician assistant feel “overwhelmed and unsure of myself.” Notably, some respondents suggested that they enjoyed their new duties at work. Some HWs reported challenges with wearing masks as part of their new routine, including difficulties breathing and providing patient care. Lastly, many HWs reported that they were grateful for being able to conduct telehealth in their home as part of their new routine. Some HWs, however, reported that there were difficulties in having meaningful patient interactions over the phone. Still, the majority suggested that telehealth was the most hopeful aspect of the pandemic. One social worker reported that being able to work via telehealth made her “have no fear of becoming infected and/or getting sick.”

The third theme within the organization level related to patient care and included the following factors: COVID-19 patient outcomes, isolated patients, non-COVID-19 medical care, and patient and family appreciation. Many HWs recounted “heartbreaking” and unprecedented experiences witnessing COVID-19 patients declining and dying. One emergency medical technician recounted a story of picking up a COVID-19 patient that was unlike any case she had seen in 30 years. “As we pulled out of the patient’s driveway, we left his entire family sitting in front of their house grieving, scared, wanting to believe there was a possibility we could save their father, knowing there was a good chance that that was the last time they would see him alive.”

On the other hand, HWs recounted specific stories of when their patients with COVID-19 improved, which gave them hope that their efforts were working. Due to hospital policies prohibiting visitors, many HWs reported that witnessing “patients die alone is traumatic to watch.” However, some HWs felt hopeful when they were able to connect with patients and their families virtually. Other HWs also reported ways in which patients without COVID-19 were negatively impacted in the hospital by the pandemic. For example, HWs reported that patients who had other diseases delayed coming to the hospital due to fear of COVID-19, which resulted in poor health outcomes. Lastly, HWs were hopeful when patients and their families expressed their appreciation for their work. One medical trainee wrote that family members’ appreciation “make[s] it worth it, and bring[s] me some hope and motivation to keep going.”

The fourth theme within the organization level related to hospital teams, including the subtheme factors of colleague diagnosed with COVID-19, teamwork, emotional support, inspiration, and supervisor relationships. First, HWs reported distress when they saw their colleagues getting treated for COVID-19 in the hospital and reported feeling “sad, angry, and scared.” Many HWs also reported upsetting experiences that related to teamwork. Most commonly, this conflict was concerning the HWs’ perception that their colleague did not want to work with COVID-19 patients out of fear of getting exposed, which made them feel “angry.” Despite these issues, many HWs reported that their most hopeful experience pertained to teamwork and having “shared experiences, a common enemy, and coming together to overcome adversity.” Many HWs also reported that they received more emotional support from their colleagues, which made them feel “closer.” HWs also shared that the inspirational, selfless work of their colleagues made them feel hopeful and proud. Lastly, some HWs suggested that getting positive feedback and appreciation from supervisors made them feel hopeful, while other HWs suggested that feeling like supervisors are not “open or sensitive to feedback” was upsetting.

Lastly, hospital management was a theme within the organization level, which included the factors of PPE policies, leadership, job security, modified schedules, and hazard pay. For example, many HWs reported that unclear, non-transparent, and transient hospital policies regarding PPE made them feel unsafe at work. One nurse even reported that his hospital’s insufficient PPE policy caused him to “never feel safe at [his] own job” and ultimately caused him to contract COVID-19. As a result, he “felt taken advantage of, disposable, and like [he] didn’t matter.” However, some respondents felt hopeful when hospital policies were changed to protect HWs. Many respondents also suggested that their hospital leadership upset them, as they did not provide open and clear communication. Other HWs that stated that their hospital did have effective leadership highlighted the importance of disseminating updates and important information to stay safe while at work. Many respondents also commented on the fear of job insecurity, which made them feel “disposable.” On the other hand, receiving hazard pay and modified schedules to decrease work hours made them feel “acknowledged.”

### Community level themes

Next, 94 (8.3%) and 175 (15.5%) HWs reported community level factors that influenced their negative and positive experiences, respectively. Five themes were present within the community level: community behavior, media, community morale, community support of HWs, and scientific knowledge of treating and preventing COVID-19.

First, many HWs reported that the community behavior during the shelter-in-place mandate contributed to their positive and negative experiences. HWs reported two factors within the community behavior theme: protesting the shelter-in-place order and compliance with COVID-19 safety precautions. For example, many HWs reported that the protests against the shelter-in-place order made them upset. Several HWs stated that the lack of social distancing and mask-wearing among community members made them “feel like our sacrifices in the frontlines (personally and to my family) were unappreciated and dismissive of patients suffering/ losses.” On the other hand, some HWs suggested that they felt “proud” that the majority of community members were compliant with the shelter-in-place order.

The second theme within the community level related to the media, including subtheme factors of misinformation and positive news. Many HWs expressed their frustration with the misinformation perpetuated by the media. HWs suggested that dealing with this “infodemic” amidst a pandemic had been “exhausting.” Some HWs reported that they felt hopeful when the media highlighted positive news stories, however.

Next, community morale was a theme at the community level and included the subtheme factors of discrimination against people of Asian descent, fear, and community altruism. For example, some HWs suggested that the “increased/blatant racism towards healthcare workers of Asian descent” within and outside of the hospital made them feel upset. Others described that the general fear in the community contributed significantly to their negative experiences. On the other hand, many HWs reported that the altruism within the community gave them hope and was “really touching.” For example, they felt hope when they heard stories of community members volunteering to deliver groceries to those in need and supporting local businesses.

Community support of HWs was another theme at the community level and included the factors of conspiracy theories, stigmatization of HWs, recognition of HWs, and donations to HWs. First, the conspiracy theories that HWs and hospitals were profiting off of the pandemic were the most upsetting aspects of the pandemic for several respondents and made them feel “so unappreciated” for their work. Others reported feeling stigmatized while in the community for being a HW, making them feel like the community is “afraid” and “disgusted” with them. However, many HWs reported that they felt support from the community through increased recognition, such as community members thanking them or doing nightly “healthcare hero” celebrations. HWs reported that donations of food and PPE to the hospitals made them “feel like we are all a team,” “like we are not alone and being seen,” and “appreciated.”

The last theme within the community level related to the scientific knowledge of treating and preventing COVID-19, and included factors of uncertainty, hope for a cure, and hope for a vaccine. HWs reported that the uncertainty of COVID-19 prevention, treatment, and how long the pandemic would last was upsetting. However, participants reported that the prospect of increased scientific knowledge to find a cure and vaccine was providing them hope.

### Public policy level themes

Overall, 56 HWs (4.9%) reported upsetting experiences and 24 (2.1%) reported hopeful experiences in the public policy level. Two public policy-level themes were present in the data: the US government and health system leadership and the shelter-in-place national and local policy. First, several respondents suggested that the US national government preparedness for the pandemic and lack of a unified response caused them significant distress. Many respondents were frustrated by the lack of scientifically driven policy to end the pandemic and thought that it was “a national disgrace.” Others reported that the misinformation being perpetuated by the national government, such as “suggesting people should inject bleach or Lysol,” made them “fearful.” One physician reported that “the lies that come out of the White House make my job more difficult and I am concerned about a resurgence when we already have had to treat 2 ICU units housing 30 COVID-19 patients.” However, a couple respondents felt hope that the pandemic would shine light on the issues with healthcare delivery in the US to spur reform.

The second theme within the public policy level related to the shelter-in-place policy and included factors of shutting down services, economic recession, flattening the curve, advocacy, and environmental benefits. First, some respondents expressed being upset that services were shut down during the shelter-in-place order, including gyms and restaurants. A few HWs reported being upset of the economic impact of shutting down services. However, many HWs reported that the shelter-in-place order was hopeful to them. Many suggested that the policy was helping to flatten the curve. Many even suggested unanticipated benefits of this policy that gave them hope, including improved environmental impact.

## Discussion

In sum, this is the first study to our knowledge that quantitatively assesses mental health morbidities among HWs in the US while also qualitatively analyzing their most upsetting and hopeful experiences during the COVID-19 pandemic. First, our study sample of HWs during the COVID-19 pandemic found that rates of probable MD and GAD in our sample were 14.0%, 15.8%, respectively, which is less than the pooled prevalence of depression and anxiety found in a meta-analysis including studies from China and Singapore (23.2% and 22.8%, respectively). The rate of PTSD in our sample (23.1%) is similar as in a study of mental health among 230 front line workers in China (27%) [30], and markedly higher than a study conducted among 470 HWs in Singapore (7.7%) [31]. No other studies to our knowledge have assessed the rate of AUD among HWs during the COVID-19 pandemic, which was over 40% in our sample. Given the variability in mental health measures, additional studies are needed in the US that validate these findings and quantitatively examine risk factors for these and other mental health morbidities.

Our study primarily analyzed responses to two open-ended prompts, which asked about HWs’ most hopeful and most upsetting experiences during the COVID-19 pandemic and how they made them feel. Our study found that on an individual level, personal health and ability to pursue self-care activities influenced their positive and negative experiences. Similarly, a review of resiliency strategies to promote HW wellbeing during pandemics concluded that promoting HWs’ ability to practice self-care on an individual level, including physical activity, sleep hygiene, and mindfulness, can foster their resiliency and decrease their psychological distress [32]. Providing HWs with resources and time to practice self-care activities and rest is vital to promote wellbeing during the COVID-19 pandemic and future outbreaks [4].

Our study also found that interpersonal factors pertaining to the health of their social circle, family functioning, and social support were important for HW wellbeing. Many HWs expressed that the fear of infecting their family was the most upsetting experience during the pandemic, which was also reported during the Middle East Respiratory Syndrome (MERS) pandemic in 2012 [33, 34]. Similar to our findings, HWs faced social isolation that negatively impacted their wellbeing during the Severe Acute Respiratory Syndrome (SARS) pandemic in 2002 [35]. In our study, HWs reported feeling appreciated, seen, and cared for when friends, neighbors, and families reached out to them, which is a reminder to the general public to continue supporting the HWs in their social circles.

Within the organization level, our study found that increased workload, new duties, difficulties providing patient care with PPE, and traumatizing patient care experiences negatively impacted HW’s experiences. Similarly, a recent qualitative study of 13 HWs who worked in COVID-19 hospitals in China found that HWs faced additional challenges in the hospital setting, namely the new context, extra workload, PPE requirements and difficult patient cases [36]. Interventions are needed that help HWs manage the increased stress at the hospital. For example, adapting a peer mentoring support program that has been historically used for soldiers in active combat could mitigate the psychological impact of these traumatizing experiences [37].

Our study also found that the lack of clear communication from the management and shortages of PPE, testing, and personnel contributed to their distress. Similarly, lack of institutional readiness to implement infection control measures, including policies and PPE distribution, impeded HWs’ ability to contain the outbreak and significantly contributed to their levels of stress and fear during the MERS pandemic [38, 39]. A systematic review and meta-analysis of 59 papers that studied the psychological effects of emerging viruses on HWs found that effective hospital leadership communication and access to PPE effectively decreased psychosocial distress [4]. Furthermore, our study findings suggest that hazard pay is an important mechanism by which hospital management can make their employees feel valued and supported.

Furthermore, we found that hospital teams can impact HWs’ wellbeing, both positively and negatively. During the MERS pandemic, increased comradery among colleagues [34, 40] and supervisor support [34, 41] were also found to improve HW morale and resiliency. Providing resources that foster team bonding and assist with conflict resolution are important to improve HWs’ experiences during the COVID-19 pandemic and future outbreaks.

Community level themes were also identified, including community morale, media, scientific knowledge of treating and preventing COVID-19, community behavior, and community support of HWs. Within the community support of HWs theme, some respondents suggested that they felt stigmatized for being a HW and were impacted by the conspiracy theories that HWs started the pandemic. Similarly, studies from the MERS and SARS pandemics found that HWs and their families experienced significant stigmatization and prejudice due to their professions, which was associated with psychological distress [35, 39, 40, 42–44]. Also similar to our findings, negative media coverage impacted staff morale during the MERS outbreak [34, 38]. However, many HWs from the present study suggested that donations of food and PPE from the community and increased recognition of HWs significantly improved their morale. These mechanisms to support HWs should thus be continued throughout the COVID-19 pandemic and future waves of outbreak.

The present study also provides important insights into novel factors impacting HW wellbeing that should be further explored. For example, the negative impact of the government and health system leadership on HW wellbeing was a theme from the data that has not yet been explored in the literature. The medical misinformation stated by political leaders was reported to make HWs’ jobs more difficult. Future studies are warranted that further ascertain the unique impact of the governmental responses on HW mental health. Ultimately, this could inform policy to improve the dissemination of scientifically driven medical information by the government to promote public health and decrease the burden on HWs. Furthermore, HW distress caused by community members’ non-compliance with COVID-19 safety precautions and belief in conspiracy theories has not been documented in the literature. Additional studies are needed that directly measure the impact of community non-compliance on HW mental health outcomes. Many HWs also reported witnessing discrimination against HWs of Asian descent during the COVID-19 pandemic. Additional studies are also needed that better understand how HWs who identify as Asian are impacted by these experiences within and outside the hospital. Lastly, the lack of childcare for HWs who have children, especially mothers, should be further explored. Many female respondents spoke about the double burden of taking care of their families while also taking care of their patients. As female HWs have been reported to have higher rates of mental health morbidities compared with male HWs during the COVID-19 pandemic [5], additional studies are warranted that explore the impact of childcare on HWs and any differences by gender or caregiver status.

### Strengths and limitations

Our study has some notable strengths and limitations. First, our sample included 1,132 HWs across all US regions who were recruited during the peak of COVID-19 transmission. The open-ended questions enabled us to learn about novel factors related to HW wellbeing that have not been studied explicitly previously. These insights can be used to further explore risk and protective factors of HW mental health during the COVID-19 pandemic and future outbreaks. Due to the anonymous nature of the survey, HWs were able to provide open and honest answers to minimize response bias. However, we were unable to analyze differences between HWs who responded and who did not respond, and our study is at risk of nonresponse bias. Our study also included significantly more females than males, and the majority of participants identified as White; future research should strive for more diverse samples, for example by oversampling racial and ethnic minority HWs. The majority of participants were also located in the Northeast, and future work should strive for better representation of HWs from other regions within the US. Yet, our study aimed to capture a range of HW positions, including physicians, nurses, health technicians/ technologists, medical assistants, medical trainees, social workers, and non-clinical workers.

In sum, this is the first study to our knowledge that has used a hybrid inductive-abductive analysis of the experiences and wellbeing of HWs during the COVID-19 pandemic in the US. Notably, we found novel factors that negatively impact HWs’ wellbeing during the pandemic that should be further explored, including national government policies, misinformation disseminated by political leaders, discrimination against people of Asian descent, and community non-compliance with social distancing. These findings situated within a Social Ecological Model can inform interventions and policies that aim to support HWs and foster their resiliency during the COVID-19 pandemic and future outbreaks.
